# Association with AflR in Endosomes Reveals New Functions for AflJ in Aflatoxin Biosynthesis

**DOI:** 10.3390/toxins4121582

**Published:** 2012-12-19

**Authors:** Kenneth C. Ehrlich, Brian M. Mack, Qijian Wei, Ping Li, Ludmila V. Roze, Frank Dazzo, Jeffrey W. Cary, Deepak Bhatnagar, John E. Linz

**Affiliations:** 1 Southern Regional Research Center, Agricultural Research Service/United States Department of Agriculture, 1100 Robert E. Lee Blvd, New Orleans, LA 70124, USA; E-Mails: brian.mack@ars.usda.gov (B.M.M.); qijian.wei@ars.usda.gov (Q.W.); ping.li@ars.usda.gov (P.L.); jeff.cary@ars.usda.gov (J.W.C.); deepak.bhatnagar@ars.usda.gov (D.B.); 2 Department of Food Science and Human Nutrition, Michigan State University, East Lansing, MI 488244, USA; E-Mails: roze@msu.edu (L.V.R.); jlinz@msu.edu (J.E.L.); 3 Department of Microbiology and Molecular Genetics, Michigan State University, East Lansing, MI 488244, USA; E-Mail: dazzo@msu.edu

**Keywords:** aflatoxin, protein trafficking, *Aspergillus parasiticus*, aflatoxin, aflatoxisomes, endosomes, AflJ, AflR

## Abstract

Aflatoxins are the most potent naturally occurring carcinogens of fungal origin. Biosynthesis of aflatoxin involves the coordinated expression of more than 25 genes. The function of one gene in the aflatoxin gene cluster, *aflJ*, is not entirely understood but, because previous studies demonstrated a physical interaction between the Zn_2_Cys_6_ transcription factor AflR and AflJ, AflJ was proposed to act as a transcriptional co-activator. Image analysis revealed that, in the absence of *aflJ* in *A. parasiticus*, endosomes cluster within cells and near septa. AflJ fused to yellow fluorescent protein complemented the mutation in *A. parasiticus* Δ*aflJ* and localized mainly in endosomes. We found that AflJ co-localizes with AflR both in endosomes and in nuclei. Chromatin immunoprecipitation did not detect AflJ binding at known AflR DNA recognition sites suggesting that AflJ either does not bind to these sites or binds to them transiently. Based on these data, we hypothesize that AflJ assists in AflR transport to or from the nucleus, thus controlling the availability of AflR for transcriptional activation of aflatoxin biosynthesis cluster genes. AflJ may also assist in directing endosomes to the cytoplasmic membrane for aflatoxin export.

## 1. Introduction

Aflatoxin is the most potent naturally occurring carcinogen of fungal origin [1,2]. Aflatoxin contamination by *Aspergillus flavus* of maize, ground and tree nuts, and cottonseed causes severe economic losses [3,4,5]. Aflatoxins exert broad toxic effects on humans. Consumption of food contaminated with aflatoxin is associated with hepatocarcinoma and lung cancer [6]. The ability to suppress immune function synergistically links aflatoxin exposure to diseases such as mSalaria and hepatitis B [7]. Exposure of children to chronic low doses of aflatoxin also may result in stunted growth, and is one of the factors linked to kwashiorkor (childhood malnutrition). Consumption of both aflatoxin and fumonisin may contribute to the HIV pandemic in Africa by affecting viral transmission and promoting disease progression [8,9]. In addition, a positive association has been demonstrated between aflatoxin exposure biomarkers in blood and anemia during pregnancy and adverse birth outcomes [10]. In some developing countries, a large portion of the population is estimated to be chronically exposed to aflatoxin through contaminated food [11,12]. 

Reduction of aflatoxin contamination of food and feed requires a comprehensive understanding of factors affecting aflatoxin biosynthesis. Aflatoxin biosynthesis is one of the most thoroughly studied secondary metabolic pathways [13]. At least 30 proteins encoded by genes clustered in a subtelomeric 75-Kb region on chromosome 3 are involved in its biosynthesis. The gene *aflR* within the aflatoxin gene cluster encodes a Zn_2_Cys_6_-type transcription factor (AflR) that is necessary for transcription of most if not all of the genes in the aflatoxin cluster [14]. This protein appears to be constitutively expressed in *A. parasiticus* but its levels and activity are controlled by developmental regulatory factors [15,16,17]. *aflJ* (also referred to as *aflS*) is divergently transcribed from *aflR* [13] and the AflJ protein was demonstrated to bind to AflR [16,18,19,20]. Because of this binding and because its disruption in either *A. flavus* or *A. parasiticus* prevented aflatoxin production, AflJ was proposed to be a transcriptional co-activator [17,18,19]. In addition, multiple copies of *aflJ* and *aflR* increased aflatoxin and aflatoxin precursor accumulation [18,21]. In *A. nidulans*, expression of *aflR* and *aflJ* homologs involved in sterigmatocystin (STC) biosynthesis is controlled by RsmA, a bZIP-type stress-response transcription factor, which binds to two AP-1-like motifs, 5'TGACACA(R)3' and 5'TTAGTAA(Y)3', located in the STC cluster *aflR*/*aflJ* intergenic region [22,23]. The *A. parasiticus* and *A. flavus aflR*/*aflJ* intergenic region lacks AP-1-like motifs and therefore regulation of expression of these genes is likely to be different in this species. Surprisingly, in an *A. parasiticus aflJ* deletion mutant (∆*aflJ*), transcripts of *pksA*, *nor*-1, *ver*-1 and *omtA* are produced, even though aflatoxin and its precursors could not be detected when the fungi were grown under conditions conducive to aflatoxin formation [17,21]. However, ∆*aflJ* failed to convert exogenously added pathway intermediates to aflatoxin, suggesting that the enzymes involved in biosynthesis are not available in Δ*aflJ* mutants [21]. 

AflJ is predicted to possess membrane-spanning domains and a microbodies-targeting signal [21]. This possible interaction with membranes suggested that AflJ may also play an additional role to its proposed role as a transcriptional co-activator. In our previous work we observed that aflatoxin is synthesized, compartmentalized, and exported via endosomes/aflatoxisomes which are generated by fusion of transport (trafficking) vesicles that carry some aflatoxin biosynthetic enzymes [24,25,26]. We presented a 2-branch model for regulation of aflatoxin gene expression and endosome/aflatoxisome development [25] where Branch 1 regulates the timing and level of expression of biosynthetic proteins in response to environmental and intracellular cues and Branch 2 regulates protein traffic and coordinates biogenesis of endosomes/aflatoxisomes which house the biosynthetic enzymes and the export of the toxin to the cell exterior by a process similar to exocytosis [26,27]. 

Since endosomes/aflatoxisomes are required for biosynthesis and eventual export of aflatoxin from the cell, and because AflJ carries transmembrane domains, we hypothesized that AflJ may reside at such organelles prior to its involvement in regulation of AflR activity. Our data suggest that AflJ binds to AflR both in endosomes and in nuclei although only AflR, and not the complex, was observed to interact with aflatoxin gene promoters. These data prompt us to hypothesize that AflJ stabilizes AflR and directs its transport to and from the nucleus and that both AflR and AflJ transit the endosome. 

## 2. Results

### 2.1. Complementation Experiments Reveal a Strong Species Specificity for AflJ Function

**Figure 1:**
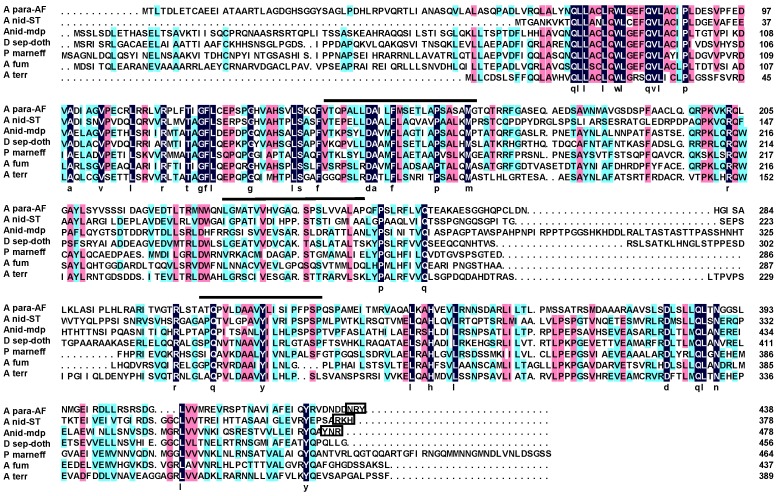
Alignment of putative AflJ homologs from different species. Amino acids marked in black are conserved in all species; whereas those marked in red or blue indicate ≥75% and ≥50% homology, respectively. Abbreviations are: A para-AF, *A. parasiticus* aflatoxin cluster AflJ AAS66019.1; A nid-ST, *A. nidulans* sterigmatocystin cluster AflJ (ANID_7819); A nid mdp, *A. nidulans* monodictyphenone cluster AflJ (ANID_10021); D. sep-doth, *Dothistroma septosporum* dothistromin cluster AflJ (R. Bradshaw, personal communication, see supplementary materials); P marneff, *Penicillium marneffei* XP_002149634.1; A fum, *A. fumigatus* XP_751378; A terr, *A. terreus* XP_001217073.1. Lines over the sequence indicate regions identified in *A. parasiticus* and *A. flavus* as membrane spanning regions. The sequences boxed at the *C*-terminal end of the AflJ sequences are putative microbodies targeting sequences [21].

AflJ belongs to a unique family of proteins found only in fungi (Figure 1). The protein is predicted to possess three membrane-spanning domains. Only AflJs from *A. parasiticus* (and other section Flavi species) and *A. nidulans* (A para and A nid in Figure 1) contain putative *C*-terminal microbodies-targeting sequences [21]. The *aflJ* orthologs that were chosen for complementation analysis are associated with gene clusters involved in sterigmatocystin and monodictyphenone gene clusters of *A. nidulans* [28], and the dothistromin gene cluster of *Dothistroma septosporum* [29] (Figure 1). *aflJ* orthologs are also found in the cercosporin gene cluster from *Cercospora nicotianae* [30] and in partial clusters from *A. terreus*, *A. fumigatus*, *Coccidioides immitis*, and *Penicillium marneffei* [31]. The *A. flavus*, *A. parasiticus*, *A. nidulans* and *D. septosporum aflJ* homologs are believed to code for proteins with a similar function in biosynthesis since the end metabolites are related to aflatoxin precursors. The function of the predicted AflJ homologs in the other species is unknown but, in each, the *aflJ* ortholog adjoins a Zn_2_Cys_6_ factor gene that resembles AflR.

**Figure 2:**
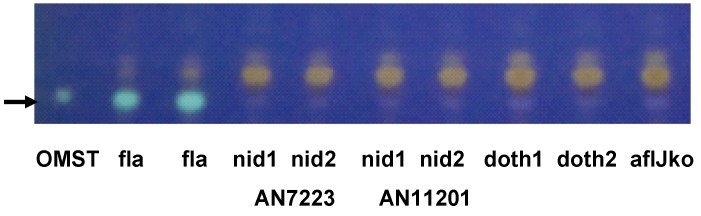
Thin layer chromatography of acetone extracts of two separate *A. parasiticus* SRRC2043 Δ*aflJ* transformants (the wild-type accumulates *O*-methylsterigmatocystin) grown on YES medium for 48 h. Complementation of *aflJ* function was tested on *A. parasiticus* Δ*aflJ* transformed with plasmid pPTRI-*gpdA*-*aflJx-trpC* where *aflJx* equals one of the tested *aflJ* gene homologs from *A*. *flavus* aflatoxin cluster (fla), *A. nidulans* sterigmatocystin cluster (nid AN7223), *A. nidulans* monodictyphenone cluster (nid AN11201), *D. septosporum* dothistromin biosynthesis cluster *aflJ* (doth, R. Bradshaw, personal comunication). The Δ*aflJ* mutant (aflJko) was included as the negative control. Two separate transformants were tested for each study. OMST is the *O*-methylsterigmatocystin standard (Sigma). Elution buffer was toluene:ethyl acetate:acetic acid (6.5:3.5:1). Cultures were extracted after 48 h growth on PDA plates.

Complementation of *A. parasiticus* Δ*aflJ* was performed with constructs carrying the following *aflJ* homologs: ANID7819 (*A. nidulans* sterigmatocystin cluster), ANID_10021 (*A. nidulans* monodictyphenone cluster), Ds-*aflJ* (*D. septosporum* dothistromin cluster (personal communication, R. Bradshaw), and AflJ (AAS90096) from *A. flavus* (positive control for complementation). Of these, only *aflJ* from *A. flavus* complemented the deletion of *aflJ* in *A. parasiticus* Δ*aflJ* as evidenced by the ability of such transformants to produce of OMST (Figure 2).

### 2.2. Aflatoxin Transcript Accumulation in Wild Type and ∆*aflJ* Transformed with aflJ from other Fungi

**Figure 3:**
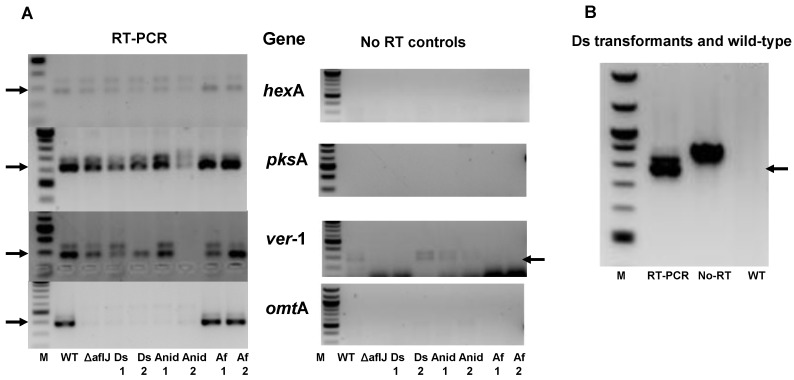
End-point RT-PCR studies. (**A**) RT-PCR was done on total RNA from fungal cultures grown on PDA medium for 48 h. The fungal cultures were *A. parasiticus* SRRC2043 Δ*aflJ* before and after transformation with the plasmid, pPTRI-*gpdA-aflJx-trpC* plasmid where *aflJx* stands for *Dothistroma septosporum aflJ* (Ds), *A. nidulans* AN7223 *aflJ* (Anid), *A. flavus aflJ* AAS90096 (Af). RT-PCR involved reverse transcription followed by PCR (oligonucleotide primers listed in Supplementary Materials) whereas the No RT controls used the same primer sets but omitted the reverse transcription step. The aflatoxin biosynthesis genes tested for expression were *hexA*, *pksA*, *omtA*, and *ver-1*; (**B**) RT-PCR was done using primers to *Dothistroma septosporum aflJ* introduced by transformation into *A. parasiticus* SRRC2043. In all cases the oligonucleotide primers sequences flanked an intron. The smaller PCR product, marked by arrows reflects amplification of the cDNA whereas the larger PCR product could be PCR from either genomic DNA or unprocessed transcript.

We confirmed, by end-point RT-PCR, the earlier study of Meyers, *et al.* [21] that some aflatoxin gene transcripts accumulated in the *A. parasiticus*-Δ*aflJ* mutant and in the transformants of Δ*aflJ* with putative *aflJ* homologs (Figure 3a). Δ*aflJ* and the other transformants expressed the aflatoxin biosynthesis genes *hexA*, *pksA*, and *ver-1* but not *omtA*. PCR studies used primer sets that flanked introns in the genes and the sizes of PCR products from cDNA were smaller than those products from amplification of DNA or unprocessed RNA. The control for these reactions was the PCR done in the absence of reverse transcriptase (Figure 3a). We confirmed that the introduced *aflJ* homologs, were processed correctly when placed under the control of the *gpdA* promoter (shown only for transformants expressing *gpdA*-Ds-*aflJ*, Figure 3b). 

### 2.3. AflJ Can Not Be Detected at Promoters of Aflatoxin Genes by Chromatin Immunoprecipitation (ChIP)

**Figure 4:**
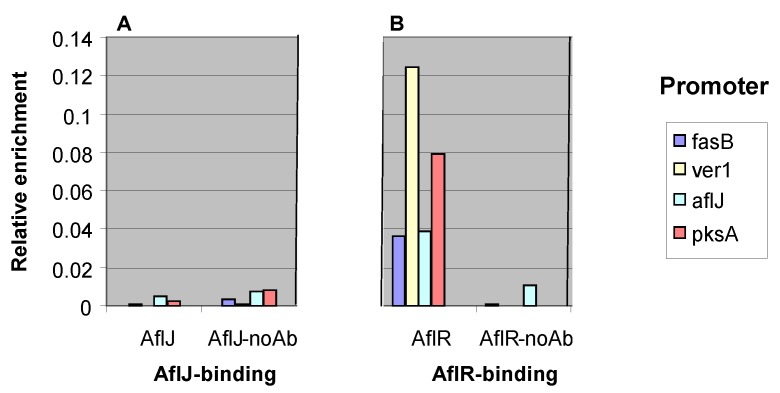
Chromatin immunoprecipitation (ChIP) to test AflJ and AflR binding to known AflR-binding sites [14]. Transformants of *A. flavus* CA14 harbored genes expressing *N*-terminal c-Myc-tagged AflR or AflJ. (**A**) Detection by PCR with oligonucleotides to either *hexB*, *ver-1*, *aflJ*, or *pksA* of chromatin DNA immunoprecipitated by anti-c-Myc in transformants expressing c-Myc-tagged AflJ; (**B**) Similar detection by PCR with the same oligonucleotides of chromatin DNA immunoprecipitated by anti-c-Myc in transformants expressing c-Myc-tagged AflR. Negative controls for both studies were DNAs isolated by ChIP in the absence of c-Myc antibody. The Y-axis (relative enrichment) is the ratio of the amount of bound DNA to input DNA given in arbitrary units.

As a transcriptional co-activator that binds AflR we expected that AflJ would co-localize with AflR at aflatoxin gene promoters that are recognized by AflR. ChIP analysis was performed on *A. parasiticus* and *A. flavus* mutants transformed with *gpdA-c-myc::aflJ* to determine if binding of AflJ could be detected at four different aflatoxin biosynthesis gene promoters. Fusion of AflJ with the cMyc epitope allowed recovery of the chromatin-bound protein by use of highly purified commercial antibodies to c-Myc. The fusion protein was used because we were unable to develop antibodies to AflJ or AflR that were suitable for chromatin immunoprecipitation. Since AflJ was previously shown to bind to AflR, we reasoned that c-Myc::AflJ would bind to AflR and allow immunoprecipitation of DNA in chromatin in promoter regions of genes from fungi grown in media conducive for aflatoxin production. As a positive control, ChIP analysis was also performed on *A. parasiticus* and *A. flavus* mutants transformed only with *gpdA-c-myc-aflR*. Although binding of c-Myc::AflR was detected at known AflR-binding sites in the *aflJ*, *pks*A, *fas*B, and *ver*-1 promoter regions (Figure 4), binding of c-Myc::AflJ to these promoters could not be detected at a level significantly different from that of the no-antibody control. 

### 2.4. Interaction of AflJ with AflR and Cellular Proteins by Yeast Two-Hybrid Assay

Using a yeast two-hybrid assay, Chang demonstrated that AflJ binds to AflR [18] and binding required essentially intact AflJ. In order to obtain additional information on possible interactions of AflJ with other *A. parasiticus* proteins, we used a 24 h *A. parasiticus* cDNA library as prey and *A. parasiticus aflJ* as bait in a yeast two-hybrid (Y2H) assay (Table 1). 

**Table 1:** Clones selected in a yeast two-hybrid experiment using *A. parasiticus* cDNA as prey and *aflJ* as bait. All yeast clones were selected on quadruple dropout media. Identification of the putative protein bound to AflJ was by BLAST search against the non-redundant fungal database at the National Center for Biotechnology Information (NCBI).

Accession number	Protein
XP_002374366	C6 transcription factor, AmdR
XP_002382582	Endonuclease
XP_002379937	AflL (AvnA)
XP_002373959	PfkA phosphofructokinase
XP_002378434	COP9 signalosome subunit 5 (CsnF)
XP_002372709	Glutamate decaboxylase
XP_001822456	COP9 signalosome subunit 5 (CsnE)
XP_002380625	FAD-dependent oxidoreductase
XP_002385336	FKBP-type peptidyl-prolyl isomerase
XP_002377941	Conidiation-specific protein (Con10)
XP_002383331	Calponin-homology-domain protein
XP_002379716	Nedd8-activating protein (UbaC)
XP_001817130	CFEM domain protein (Pfam03750)
XP_001817064	SteA
XP_003190896	Duf 3752 domain protein (Pfam 12572)
XP_001727088	MFS superfamily (maltose permease)

Some of the clones selected in the Y2H experiment are predicted to encode proteins associated with components of the COP9 signalosome and a putative Nedd8-activation. Others detected in the assay, including Con-10 and SteA, are proteins known to be involved in developmental processes in fungi, while another (AflL) is a cytochrome P450, a type of enzyme typically associated with membranes in peroxisomal organelles. Although AflR was not found among the interacting clones, a different Zn_2_Cys_6_ factor AmdR was detected. When tested separately using yeast transformants expressing AflR as bait and AflJ as prey as was done by previously [18], we were able to confirm the ability of these proteins to interact as demonstrated by the formation of yeast colonies on selective medium (Table 2). 

**Table 2:** Yeast colonies selected on quadruple dropout medium (QDO) after co-transformation with binding domain plasmid (BD) and activation domain plasmid (AD) domain plasmids, AflJ and AflR. Full-length coding sequences for AflJ or laminarin (negative control) were inserted into pGBKT7, BD) and either AflJ or AflR were inserted into the (pGADT7, AD). Selection was on yeast minimal medium plates lacking Trp, Leu, His, and Ade (QDO) as described in the Clonetech Matchmaker Kit (see Experimental Section).

BD	AD	Colonies
AflJ	AflR	125
AflJ	AflJ	0
AflJ	pGADT7	0
Lam	AflR	0
Lam	AflJ	0

Split enhanced yellow fluorescent protein (eYFP) assays (see below) also confirmed that AflJ is able to bind to AflR. Taken together, these results suggest that AflJ, besides binding to AflR, is able to bind to proteins associated with peroxisome-like vesicles. 

### 2.5. AflJ-GFP Localizes Mainly to Endosome-Like Cellular Structures

**Figure 5:**
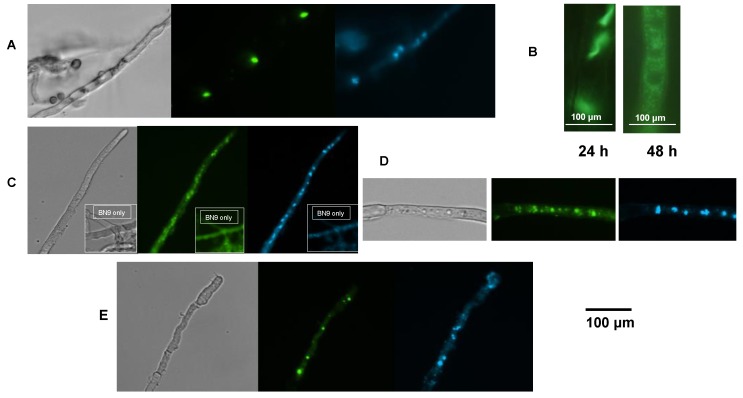
AflJ and AflR localization in *A. parasiticus* of GFP- and eYFP-tagged fusion proteins. (**A**) AflJ::GFP localization: expression of the fusion construct was under the control of the *gpd*A promoter. Mycelia were obtained from cultures of transformants of *A. parasiticus* Δ*aflJ* with pPTRI-*gpdA-GFP::aflJ*-*trpC* grown on PDB medium for 24 h. The mycelia was stained with DAPI and examined for fluorescence using a GFP and a DAPI filter; (**B**) AflJ::GFP localization when the fungi were grown on YES medium for 24 and 48 h; (**C**) AflJ::GFP localization when expression of the fusion protein was under control of the *aflJ* promoter. Fluorescence was determined on *A. parasiticus* mycelia when the fungus was transformed with pPTRI-*aflJ* promoter*-GFP::aflJ-trpC* terminator grown on PDB for 18 h. Insets show self-fluorescence and DAPI-fluorescence when the wild-type *A. parasiticus* BN9 was examined in the microscope under the same conditions; (**D**) AflR::GFP localization. Fluorescence was determined on *A. parasiticus* transformed with *gpdA-YFP::aflR-trpC* grown as above; (**E**) AflJ and AflR split YFP (BiFC) studies. Fluorescence was determined on mycelia obtained from *A. flavus* co-transformed with plasmids *amyB-aflR::Nt-YFP* and *amyB-aflJ::Ct-YFP*. All micrographs were acquired at 400× final magnification.

Plasmids containing *aflJ* fused to the jellyfish green fluorescent protein *(aflJ-GFP*) were transformed into *A. parasiticus* Δ*aflJ*. These were used to investigate the intracellular localization of AflJ in live *A. parasiticus* cells. The GFP constructs were prepared with either the glycerol phosphate dehydrogenase (*gpdA*) or the *aflJ* promoter driving gene expression. Images of the intracellular localization of AflJ-GFP in live hyphae were taken at various times after growth in PDB liquid medium (Figure 5). At 24 h, large fluorescent structures were observed in the cytoplasm. These large structures seem to represent protein aggregates (Figure 5a). Generally, these fluorescent aggregates do not co-localize with DAPI-stained nuclei. At later times (48 h) AflJ-GFP localized both to aggregates and to small dot-like structures reminiscent of endosomes in approximately 30% of examined cells (Figure 5b). When the *aflJ* promoter was used to drive *aflJ* expression, most of the green fluorescence still co-localized with endosomes rather than with the DAPI-stained (blue fluorescent) nuclei (Figure 5c). 

### 2.6. AflR-GFP Localization

A similar fusion construct was created with the *gpdA* promoter driving expression of *aflR* fused to *GFP*. *A. parasiticus* transformants containing this plasmid also showed fluorescence in cellular aggregates that were similar to those seen in the AflJ-GFP fusion studies (Figure 5d). Both discreet fluorescent aggregates and smaller dots consistent with endosomes were visible in the mycelia. The majority of the GFP fluorescent signal did not appear to co-localize with the DAPI-stained nuclei.

### 2.7. Bimolecular-Fluorescence Complementation (BiFC, Split eYFP) Assays

Because previous studies using the yeast two-hybrid assay concluded that AflJ interacts with AflR in nuclei [18], we investigated the interaction of AflJ with AflR using a split eYFP assay. Transformants containing both the amylase gene promoter (*amyB*) driving expression of the *N*-terminal half of the eYFP coding sequence fused to *aflR* (*amyB-Nt-eYFP-aflR*) and the *amyB* promoter also driving expression of the *C*-terminal half of the eYFP coding sequence fused to *aflJ* (*amyB-Ct-eYFP-aflJ*) were grown in either maltose (inducing) or glucose (non-inducing; data not shown) minimal media. Only induced co-transformants showed Ct-eYFP::AflJ and Nt-eYFP::AflR interaction as evidenced by fluorescence within small and large dots consistent with vesicles and endosomes within the mycelia (Figure 5e) similar to those observed for the AflR-GFP transformants. Only a few of the dots (the smaller ones mainly) co-localized with DAPI-stained nuclei when the cultures were grown on minimal media containing maltose. 

### 2.8. AflJ Is Associated with Endosome Biogenesis and Traffic

To investigate if AflJ is involved in endosome/aflatoxisome biogenesis we examined wild-type and Δ*aflJ A. parasiticus* cultures grown for 44 h or 68 h cultures using bright field microscopy. At both time points, wild-type cultures, grown similarly, produce aflatoxins. We hypothesized that disturbance of endosome phenotype could indicate changes in biogenesis of endosomes and/or trafficking of endosomal proteins that are involved in aflatoxin biosynthesis. As part of the analysis, parameters such as the number, size, timing of appearance, and sub-cellular localization of structures consistent in size and shape with endosomes were characterized. In previous work we obtained biochemical and genetic evidence that these structures are endosomes [25].

**Figure 6:**
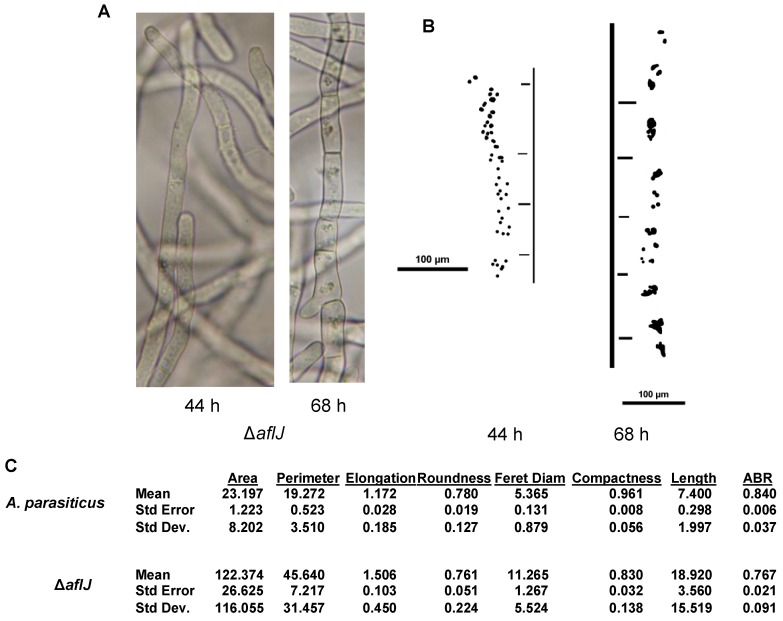
Comparison of vesicle/endosome distribution in the *A. parasiticus* Δ*aflJ* mutant grown in the dark for 44 h and 68 h on YES liquid medium. (**A**) Morphology of Δ*aflJ* was evaluated at indicated time points using bright field microscopy; (**B**) Binary images of the corresponding distribution of vesicles and endosomes in wild-type *A. parasiticus* and Δ*aflJ*; (**C**) Descriptive statistics of size and distribution of the vesicles/endosomes in Figure 5B measured by CMEIAS digital image analysis [32]. The measurement features are defined in Supplementary information.

At 44 h in wild-type *A. parasiticus*, endosomes were just starting to form; they were small and were present in <10% of the cells (data not shown), whereas in the Δ*aflJ* mutant, at this same time, nearly 50% of the cells carried distinct endosomes (Figure 6a,b). By 68 h, larger more elongated clusters (Figure 6c) of organelles were observed in more than 50% of the cells from both the wild-type and the mutant. In the wild-type these appeared mainly as discreet units. However, for a small number of examined mycelia, endosomes were detected in small clusters located primarily near a septum. The number of endosomes per cluster ranged from 3 to 6, and the number of endosomes per cell ranged from 30 to 46 (Figure 6c). In Δ*aflJ*, by 68 h, large clusters of organelles were observed in 75% of cells and were located near septa, as in the wild type, and in the middle portions of the cells. The number of endosomes per cluster ranged from 5 to 14. 

## 3. Discussion

Several lines of evidence presented in this work suggest that AflJ performs a specialized function in cellular organization that affects AflR activity and aflatoxin biosynthesis. We previously developed a comprehensive model based on aflatoxin biosynthesis, to illustrate current understanding of the biosynthesis of hydrophobic secondary metabolites in fungi and plants [24,25,27]. This model proposes that secondary metabolite biosynthesis is a spatially organized, multistep process that occurs in dedicated sub-cellular organelles within the cell. Specifically, aflatoxin biosynthesis was demonstrated to involve an intricately coordinated process where synthesis occurs primarily in endosomes/aflatoxisomes and export of the toxin occurs when the aflatoxisomes fuse with secretory vacuoles. The model suggests that biosynthesis of secondary metabolites requires the appropriate timing and level of expression of the biosynthetic enzymes in response to environmental and intracellular cues. These enzymes are then mobilized by specialized vesicles and transported to endosomes/aflatoxisomes where they interact to catalyze toxin synthesis. 

The real-time sub-cellular localization data demonstrate that AflJ and AflR fusion proteins co-localize in the cytoplasm in organelles that are consistent in size and shape with endosomes as well as in the nucleus supporting the hypothesis that AflJ binding to AflR may function in several aspects of aflatoxin biosynthesis regulation. Localization in endosomes is supported by the images in Figure 5 since the fluorescence appears to be mainly associated with aggregated vesicles rather than vacuoles. Vacuoles in the mycelia have a distinctly different appearance from endosome-like organelles and from nuclei and stain with the MDY64 stain while endosomes do not (see Figure S7). Also, localization in endosomes is consistent with a previous study in which AflR was detected in endosomes [27].

In the *aflJ* mutant some of the early aflatoxin biosynthesis genes are still expressed while the transcript of at least one of the later genes (*omtA*) cannot be detected (Figure 3). ChIP analysis failed to detect AflJ binding at known AflR cis-acting sites for certain early aflatoxin biosynthesis genes, but this result does not rule out the possibility that AflJ might be necessary for transcription of later biosynthesis genes such as *omtA*. It is also possible that, in the absence of AflJ, which we suggest facilitates trafficking of AflR to the nucleus, an insufficient amount of AflR is available for activation of genes involved in the later steps of aflatoxin biosynthesis. Based on AflJ’s proclivity to interact with COP9 signalosome components as demonstrated by yeast two hybrid analyses, by forming a complex, AflJ may stabilize AflR within the endosome. This role might be similar or alternative to the role of proteins in endocytic recycling found in most eucaryotes [33]. Alternatively, AflR bound to AflJ and delivered to endosomes/aflatoxisomes may represent a membrane-tethered transcription factor poised for activation and transport to the nucleus. In Arabidopsis membrane-tethered transcription factors are involved in stress-response and developmental regulation [34,35,36]. 

Our data indicate that AflJ possesses a strong propensity to aggregate. Large aggregates of AflJ-GFP fusion protein were found in fungal cells especially when *aflJ* was expressed under control of the *gpdA* promoter where unnaturally high amounts of AflJ are presumably being produced. Although, it is possible that the localization results only reflect such high amounts of protein, controls done with two proteins known to interact in the nucleus, *laeA* and *veA,* when expressed as fusion proteins to the *N*-terminal and *C*-terminal halves of eYFP and under the control of the strong *amyB* promoter, mainly localized to nuclei, as expected [37], whereas the non-interacting pair, *C*-terminal eYFP-*veA* and *N*-terminal-eYFP-*aflR* showed no detectable fluorescence in organelles or in the nucleus (see Figure S6). Also, we found that *aflJ-GFP* complemented the *aflJ* deletion and toxin production was restored, but at lower yields than were found in the wild-type strain. The strong ability of AflJ to aggregate was predicted by AGGRESCAN, a web based software for the prediction of aggregation-prone segments in protein sequences [38]. Using this program we identified 17 “hot spots” of aggregation in AflJ based on “hot spot” distribution in different protein groups presented by Conchillo-Sole *et al*. [39]. In contrast, GFP contains only 6 “hot spots”, and AflR possesses 11 “hot spots”. Intriguingly, five of the “hot spots” detected in AflJ overlap the predicted three membrane-spanning domains (aa 139–164, 232–252, 306–326; Figure 1) identified by Meyers *et al*. [21]. In addition, the *C*-terminal region of AflJ, which was identified as not being critical to AflJ’s binding to AflR [18], was not associated with any of the aggregation “hot spots”. We suggest that the aggregation propensity of AflJ may explain the association of this protein with the specific cellular compartment where it resides. We also presume that the increased production of AflJ may be responsible for its formation into cytoplasmic aggregates. Such aggregates also were present when *aflJ* and *aflR* were co-expressed under control of the *amyB* promoter, but smaller numbers of aggregates were detected when these proteins were expressed under control of their own promoters. 

Microscopic analysis and ChIP data, while not disproving the function of AflJ as a transcriptional co-activator, suggest an alternative function for AflJ in the control of AflR activity. Previous studies showed that the ratio of AflR to AflJ may be important for controlling AflR’s ability to activate genes in the aflatoxin cluster [16,40]. When the ratio of *aflJ* to *aflR* transcript was < 1 due to either an unfavorable water activity or temperature stress, the production of aflatoxin was low, whereas when the ratio was > 1, for example at temperatures or water activities conducive to aflatoxin formation, production of aflatoxin was high. This relationship could explain the differences in results obtained in different studies recently published which compared *aflJ* and *aflR* expression at 37 °C and 30 °C [16,40,41]. One study found that the ratio of *aflR* to *aflJ* is around 1.0 at both 30 °C and 37 °C [41], whereas another study found that the ratio of *aflR* to *aflJ* is about 0.3 at 30 °C while the ratio is 0.1 at 37 °C [40]. In the latter study it was proposed that high levels of AflJ are needed to overcome a repressor of *aflR* transcription [40]. Although evidence for an aflatoxin biosynthesis repressor has been reported [42], such a protein has not yet been isolated.

Consistent with our results, we suggest that AflJ acts as a chaperone-like protein to promote transit of AflR from the endosome/aflatoxisome to the nucleus and perhaps back to the endosome/aflatoxisome for eventual degradation. If AflJ is not expressed at sufficient levels to protect AflR and act in its transport, the intact protein may not able to reach the nucleus in sufficient amounts to turn on the many genes required for aflatoxin biosynthesis. Chang *et al*. reported that AflJ binds to AflR through two *C*-terminal domains in AflR and that most of AflJ sequence is required for the interaction [18]. We propose that AflJ may serve to tether AflR to the endosome/aflatoxisome membrane and thereby, to modulate transcription factor availability in the nucleus. The results from the yeast two-hybrid assay are consistent with AflJ helping to stabilize AflR by binding to factors that control protein degradation such as Nedd8 and CsnE and CsnF, components of the COP9 signalosome. The COP9 signalosome had been found to control production of a factor that also localized to cellular organelles to affect conidial development [43]. 

In the fungal cell, when aflatoxin synthesis is initiated, endosome fusion to the vacuole is down-regulated by the global regulator VeA resulting in the accumulation of endosomes/aflatoxisomes [44]. Then, endosomes/aflatoxisomes that carry aflatoxin, move to the cytoplasmic membrane and dock there to dump their contents into the surrounding growth medium. In the absence of AflJ, docking to the cytoplasmic membrane appears to be blocked, thereby resulting in endosome clumping within the cell. It is possible that AflJ, besides its presumed role in AflR transport, is also required for maintenance of the proper membrane structure of the aflatoxisome and, therefore, may affect the export of aflatoxin by the aflatoxisome. The failure to properly remove aflatoxin from the cell could have a feedback inhibitory effect on aflatoxin gene transcription consistent with the decreased levels of transcripts observed in Δ*aflJ*.

## 4. Experimental Section

### 4.1. Strains, Media, and Growth Conditions

*A. parasiticus* SU-1 (ATCC 56775) and *A. parasiticus* BN009E (BN9) [45] were used as wild type aflatoxin producing species and for preparation of transformants for the split eYFP (BiFC) studies, *A. parasiticus* SRRC2043::Δ*aflJ* (ApΔ*aflJ)* [18] was used for complementation studies and preparation of some of the transformants in which *aflJ* or *aflR* was fused to GFP coding sequence. For the ChIP studies *A. flavus CA14 Ku70^−^* [46] was used to prepare the transformants. Cultures were maintained on V8 agar (5% V8 juice/2% agar) [47] and grown on YES (2% yeast extract and 6% sucrose, pH 5.8) or potato dextrose agar (PDA) or broth (PDB) liquid medium for production of aflatoxin or its precursor, OMST. Maltose was substituted for glucose in A&M-medium for growth of transformants expressing the split eYFP fusion proteins. Conidiospores (spores) were inoculated into liquid growth medium at approximately 10^4^ spores per mL and incubated for appropriate time periods at 30 °C with shaking at 150 rpm in the dark (standard growth conditions).

### 4.2. Chromatin Immunoprecipitation (ChIP)

For ChIP analysis, each recombinant protein was tagged at the *N*-terminal end with a c-Myc epitope tag (EQKLISEEDL) by first cloning the gene into the Clontech vector pGBKT7 followed by recovering the tag and gene by PCR with appropriate primers. After recloning the PCR product into pPTRI-gpd::trpC [48] and transforming into *A. flavus* CA14, ChIP was performed on mycelia grown on PDB for 18 to 90 h, using a modification of the procedure outlined in the product brochure for Millipore EZ ChIP™ (Millipore, MA, USA). Mycelia were treated with formaldehyde (final concentration of 1%) for 10 min and cross-linking was terminated by adding glycine to a final concentration of 0.125 M. After rinsing with phosphate buffered saline (PBS), the mycelia were ground to a powder in liquid nitrogen. Ground mycelia (100 mg) was suspended in 1 mL SDS lysis buffer (1% SDS, 10 mM EDTA and 50 mM Tris, pH 8.1), PMSF (1:100 of 0.125 M stock in iso-propanol) and protease inhibitor cocktail (1:100; Sigma-Aldrich, St Louis, MO, USA; catalog number P8215). The suspension was sonicated (Sonics Vibracell Newtown, CT, USA; catalog number VCX130) with the 3 mm microtip on a freezer block at an amplitude of 25% for 4 pulses of 30 s and a 30 s rest between pulses. The average product size after sonication was 300 to 400 bp. After centrifugation for 10 min at 14000 × *g* at 4 °C, the supernatant (200 μL) was diluted with 1.8 mL ChIP Dilution Buffer (0.01% SDS, 1.1% Triton X-100, 1.2 mM EDTA, 16.7 mM Tris-HCl, pH 8.1, 167 mM NaCl with PMSF and protease inhibitors to 200 μL). From each sample 2 μL was saved for determination of the concentration of input chromatin DNA. Before immunoprecipitation, the sample was precleared by adding 80 μL salmon sperm DNA/Protein G Agarose mixture (Millipore, Billerica, MA, USA) and incubating on a rocker platform for 1 h at 4 °C. After centrifugation for 1 min at 5000 × *g* to remove beads 5 μL anti-c-Myc (abcam ab9132) was added to samples and incubated overnight at 4 °C. A sample was also incubated identically without including the antibody. Detection was with anti-c-Myc (abcam Eugene OR, ab9132) antibody (Ab) by incubation overnight at 4 °C. The negative control was the sample with no Ab added. Chromatin bound to c-Myc tagged protein was precipitated by adding 60 μL salmon sperm DNA/Protein G Agarose beads for samples that received anti-c-Myc Ab. Incubation was for 1 h at 4 °C. The beads were harvested by centrifugation at 1000 rpm for 1 min and washed for 4 min at 4 °C with 1 mL of Low Salt Immune Complex Wash Buffer, High Salt Immune Complex Wash Buffer, LiCl Immune Complex Wash Buffer (reagents supplied with a Kit from EMD Millipore, Darmstadt, Germany), and TE Buffer (2 washes). The beads were then re-suspended in 100 μL elution buffer (50 mM Tris-Cl, pH 7.5, 10 mM EDTA, 1% SDS) and incubated for 10 min at 65 °C, centifuged as done previously, and the supernatant collected. The extraction was repeated once and 20.8 μL of 5 M NaCl (200 mM NaCl final) and 1 μL proteinase K (20 mg/mL stock) were added. The solution was first incubated at 45 °C for 2 h and then at 65 °C overnight to reverse crosslinks. The DNA was purified using a QIAquick PCR purification kit (Qiagen, Valencia, CA, USA) and qPCR was performed using 2 μL of the 100 μL recovered DNA as the template. qPCR was conducted on the recovered DNA and input DNA using the Power Sybr Green Kit (Applied Biosystems, Carlsbad, CA, USA; Catalog # 4367659) and 40 cycles of amplification. qPCR determinations followed the guidelines suggested by Bustin, *et al*. [49]. Oligonucleotides were designed using Vector NTI to give amplicons ranging from 100 to 150 bp (Table 1). Primer efficiency for DNA amplification was determined to be 80% to 110%. 

### 4.3. Complementation Studies

The coding sequences of putative *aflJ* homologs from *A. nidulans* and *D. septosporum* were PCR-amplified from genomic DNA of these species using oligonucleotide primers to the *N*- and *C*-terminal coding sequences of each gene. After cloning into pPTRI-gpdA-trpC, *A. parasiticus* Δ*aflJ* was transformed with the resulting plasmid DNA as previously described [48], and pyrithiamine-resistant transformants selected. The ability to produce OMST by these transformants was assessed by thin layer chromatography (TLC) on Baker, Phillipsburg, NJ Silica250 plates after elution with toluene:ethylacetate:acetic acid (6.5:3.5:1). 

### 4.4. Yeast Two-Hybrid Analysis

Yeast two-hybrid studies were performed using the Matchmaker Library Construction and Screening Kit (Clontech, Mountain View, CA, USA). *aflJ* from *A. parasiticus* BN9 was used as the bait by insertion of the gene into plasmid pGBKT7 while cDNA from BN9 grown for 48 h on PDA medium was introduced into pGADT7-Rec to generate the GAL4 activation domain plasmid. All procedures were conducted according to published Matchmaker protocols. Selection of *Saccharomyces cerevisiae* AH109 colonies was achieved by growth for 5 days at 30 °C on SD/-Ade/-His/-Leu/-Trp (quadruple dropout, QDO) agar. Colonies were analyzed by PCR using the Matchmaker 5' and 3'AD LD-insert screening primers specific to pGADT7. The PCR products were sequenced and the sequences analyzed by BLAST analysis of the non-redundant DNA database in GenBank as well as against the *A. flavus* NRRL3357 sequenced genome.

### 4.5. Fluorescence Microscopy and Split eYFP Studies (Bimolecular Fluorescence Complementation-BIFC)

For subcellular localization of AflJ and AflR at different time points after germination, *A. parasiticus* Δ*aflJ* transformants with pUC18-*gpdA* promoter-*aflJ::GFP*-*nmt1* terminator or pUC18-*gpdA*-*aflR::GFP*-*nmt1* were prepared, where *aflR*::GFP and *aflJ*::GFP are in-frame fusions of the coding sequences for *aflJ* or *aflR*, and the coding sequence for the enhance green fluorescent protein modified from *Aequorea victoria*. For split eYFP analysis [37], the vectors used to introduce *aflR* and *aflJ* were pMCB17apx-*amyB*-Ct-eYFP-*trpC* and pPTRI-*amyB*-Nt-eYFP-*trpC*. The former plasmid contains the maltose-inducible *amyB* promoter [50], and the *C*-terminal (Ct) portion of eYFP followed by the *A. nidulans trpC* transcriptional terminator, while the latter plasmid contains the *amyB* promoter, the *N*-terminal portion of eYFP and the *trpC* terminator. *aflR* was inserted in-frame after the Nt-eYFP sequence while *aflJ* cDNA was inserted in-frame after the Ct-eYFP sequence. The two plasmid constructs were cotransformed into *A. flavus CA14 pyrG*Δ,*Ku70*Δ and transformants containing both vectors were selected by growth on Czapek’s medium containing 0.1 μg/mL pyrithiamine. The negative control for the split eYFP assays was a cotransformant containing Nt-eYFP-*aflR* and Ct-eYFP-*veA*. The positive control was a cotransformant containing Nt-eYFP-*laeA* and Ct-eYFP-*veA* [37]. Transformants were grown on minimal media with either glucose (non-inducing) or maltose (inducing) as the sole carbon source. Fluorescence was examined on a Nikon, Melville, NY, Eclipse E600 microscope with either a Endow GFP HQ filter cube (Ex 450–490, DM 495, BA 500–550 or a Yellow HYQ filter cube (EX 490–510, DM 515, BA 520–550). A 10× eyepiece and a 40× objective were used for most microscopy. Nuclei were stained with 1 μg/mL DAPI (4',6-diamidino-2-phenylindole) and examined using the UV-2E/C filter cube (EX 330-380, DM 400, BA 435-485). For bright-field microscopy, hyphal filaments were observed under a Nikon Eclipse E600 microscope. Computer-assisted image analyses of endosomes in wild-type and Δ*aflJ* *A. parasiticus* were performed using CMEIAS^©^ Ver. 1.27 (Center for Microbial Ecology Image Analysis System, MSU, MI, USA) [32].

## 5. Conclusions

Our work provides new insights into the function of AflJ, supporting a new role as a key endosome/aflatoxisome protein that may be involved in aflatoxin biosynthesis by mediating both the availability of AflR for transcriptional activation and the biogenesis and transport of aflatoxisomes/endosomes. The data confirm previous observations that AflR and AflJ interact physically and that, while AflR participates in a stable complex in aflatoxin gene promoters, AflJ does not. We do not rule out the possibility that AflJ transiently interacts with these same promoters. The data suggest that AflJ interacts with peroxisomal membrane proteins, a role consistent with presence of several transmembrane domains in the AflJ protein. The data strongly suggest (but do not confirm) an alternative role for AflJ to its previously suggested role as a transcriptional co-activator. The data strongly suggest that AflJ interacts with AflR in organelles consistent in size, shape, number, and staining characteristics with endosomes. Our previous study [27] provided supporting evidence that AflR interacts with endosomes. Based on these data, we propose a hypothesis that AflJ chaperones AflR, that these proteins transit the endosomes on the way to the nucleus, but that predominantly AflR is an important part of the transcription complex. Preliminary data on endosomes in Δ*aflJ* suggest that AflJ may also play a secondary role in eventual localization of endosomes to the cytoplasmic membrane during exocytosis.

## Supplementary Files

Supplementary File 1 Supplementary Information (PDF, 1011 KB)
