# Supplementary material for: Association with AflR in Endosomes Reveals New Functions for AflJ in Aflatoxin Biosynthesis
Source: Toxins (Basel). 2012 Dec 19;4(12):1582–600. doi: 10.3390/toxins4121582 (PMC3528264; doi:10.3390/toxins4121582)
Supplement: Supplementary File 1 — Supplementary Information (PDF, 1011 KB) [file toxins-04-01582-s001.pdf]

Supplementary Information

Table S1. Fungal strains and transformants.

Aspergillus parasiticus SU1
Aspergillus parasiticus BN9
Aspergillus parasiticus RHΔ*aflJ*, *ptrA*$^{-}$
Aspergillus parasiticus RHΔ*aflJ-gpd-A. flavusaflJ*
Aspergillus parasiticus RHΔ*aflJ-gpd-D. septosporumaflJ*
Aspergillus parasiticus RHΔ*aflJ-gpd-A. nidulans ANid7819aflJ*
Aspergillus parasiticus RHΔ*aflJ-gpd-A. nidulans ANid10021aflJ*
Aspergillus parasiticus RHΔ*aflJ ptrA*$^{+}$ *gpdA-aflJ::GFP*
Aspergillus parasiticus BN9 *ptrA*$^{+}$ *gpdA-aflR::GFP*
Aspergillus flavus CA14 Δku70 ΔpyrG-*ptrA*$^{-}$
Aspergillus flavus CA14 Δ*ku70* Δ*pyrG-ptrA*$^{+}$*-gpdA-c-myc::aflJ*
Aspergillus flavus CA14 Δ*ku70* Δ*pyrG-gpdA- ptrA*$^{+}$*-c-myc::aflR*
Aspergillus flavus CA14 Δ*ku70* Δ*pyrG-amyB-Ct-eYFP::aflJ*
Aspergillus flavus CA14 Δ*ku70 amyB-Nt-eYFP::aflR*
Aspergillus flavus CA14 Δ*ku70 ptrA*$^{+}$*-amyB-Nt-eYFP::aflR; amyB- Ct-eYFP::aflJ*

Table S2. Oligonucleotides used for QPCR [a].

**Quantitative PCR in chromatin immunoprecipitation assays**		
**Name**	**Location**	**Sequence**
pksA-F	15865	TTCGTCGTTGACCGATGAGCTGAGT
pksA-R	16007	CGATGGCCACATGGTGCAATAA
nor-1-F	17347	AACTCGGCCAGCGACCAACACA
nor-1-R	17484	GCCTCTCTTGATCGTGCTGGCTAA
fasB-F	25282	ATCGGTTCAATGCTCGAACACCTAA
fasB-R	25413	CAGCCTGGCTGCCATTCTTGA
aflJ-F	34810	GGGACGTTCAGTAGCTCTCCTTGCA
aflJ-R	34938	GGCGCAGGTTTCTAGGTCAGTCA
ver-1-F	41839	CGCCGCCCGATGAGCTACTGGT
ver-1-R	42100	GACGGCGATGGCAGCACCGA

[a] Location is in *A. parasiticus* aflatoxin cluster sequence; GenBank Accession number AY371490. Oligonucleotide sequences are written from the 5' to 3' direction. Oligos designed using designed using Primer Express3, Applied Biosystems.

Table S3. PCRs to prepare DNAs used for aflJ gene complementation.

**Name**	**Sequence**
AF-aflJ-Not-F	aatagcggccgcATGACCTTGACTGACCTAGAAACC
AF-aj-Rsr-R2	aggctcggaccgGGCGTGATAGAGTCTTCCGGCTA
Nid-7819-NF	actagagcggccgccaccATGACCGGTGCTAACAAAGTAA
Nid-7819-CR	aggctcggaccgTTAGTGCTTTCTAGCAGACGGTTC
Nid-10021-NF	actgtagcggccgccACCATGTCTAGTCTATCCGA
Nid-10021-CR	gtcgcacggtccgatTCACCTGTTATAGGCCTGGT
Doth-aj-FNot	actgtagcggccgcCACCATGTCGCGGATCTCT
Doth-aj-RKpn	aggcatggtaccTCATCCTAAGAGCTGCGGCTG

Plasmids were created in pTRI-*gpdA-trpC* [4] by cloning into NotI and RsrII or SmaI sites.

Table S4. Oligos to check for expression of *A. parasiticus* aflatoxin cluster genes.

Name	Sequence	DNA product size	RT-PCR size
pksA-F	ATGGCTCAATCAAGGCAACT	756	625
pksA-R	CTTAGAGATGCAGCTGATCCAC	-	-
fasA-F2	CAAGAATGCCATCTTTGTTGAG	331	281
fasA-R2	CGCCATAGCTGATGCTCA	-	-
omtA-F	ACAGGATATCATTGTGGACGG	465	387
omtA-R	ATACCTAGATCAAAGCGGCG	-	-
Ver-1 F2	GGATCCTGAGGCAACTGC	331	281
Ver-1 R2	CCTTGGACCCGGAGTATACA	-	-
Doth-F	AGAAGTGTTGCTGGCGGAA	949	854
Doth-R	GTCGCAGAATGTAGACGGCT		-

fasA, *pksA*, *ver-1*, *omtA* in *Aspergillus parasiticus* SRRC2043-Δ*aflJ* complemented with *gpd-A. flavus aflJ*; *gpd-D. septosporum aflJ.*

Table S5. Oligos for preparation of constructs used for Y2H studies.

Name	Sequence
AflR-F (*NdeI*)	gagcatatgGTTGACCATATCTCC
AflR-R (*BamHI*)	attggatccCATTCTCGATGCAGGTAAT
AflJ-F (*NdeI*)	cagcatatgACCTTGACTGACCTAGA
AflJ-R (*BamHI*)	attggatccTTATATCGGTTGTCAT

Table S6. Oligos for cloning into pTRI-*gpd-trpC* for expression of AflJ with myc tag.

Name	Sequence
pgb-cmyc-F (*NotI*)	gagcatatgGTTGACCATATCTCC
aflJ-R (*RsrII*)	gcattcggtccgTTATATCGGTTGTCATTG
aflJ-R (*SmalI*)	TCATTCTCGATGCAGGTAAT

Table S7. Oligos for cloning into puc18-*gpdA-eGFP-nmt1* for expression of AflR and AflJ with the GFP tag.

Name	Sequence
AflJ-F (*NcoI*) (A.p.)	CGAGCCATGGCCTTGACTGACCTAGA
AflJ-R (*NcoI*) (A.p.)	GCATTCCATGGcATATCGGTTGTCAT
aflR-InFusion*-pUC-F	GCAGACATCACCATGGATGGTTGACCATATC
aflR-InFusion*-pUC-R	CCCTTGCTCACCATGGcttctcgatgcaggta
aflR-aflJ-InFusion*-F	GCATGATGGCCGTCATGCATTCATTCTCGATGCAGGTA
aflR-aflJ-InFusion*-R	CCCTTGCTCACCATGGCATATCGGTTGTCATC

* InFusion is a non-restriction enzyme method for cloning developed by Clonetech.

Figure S1. Plasmids used for the fungal transformations. (**A**). pTRI-*gpdA-trpC* was derived from pPTRI a cloning plasmid available from Takara with the A. oryzae ptrA (pyrithiamine resistance gene) for selection on pyrithiamine containing media. This plasmid was used for construction of the expression plasmids for the aflJ homologs; (**B**) This pUC-based plasmid was used for construction of the expression plasmids for the *aflJ* homologs and for preparation of the *aflJ::GFP* and *aflR::GFP* plasmids Introduction into *Aspergillus* was by co-transformation with Pptri; (**C**) pTRI-*gpd-trpC* was used to create the *N*-terminal split YFP vector, where the gpdA promoter was replaced by *amyB* promoter and Nt-*eYFP-aflR* was introduced by overlap PCR from a plasmid first created in pMCBapx; (**D**) pMCB17apx-amyB was used as the plasmid for construction of the *C*-terminal split eYFP vector. The gpdA promoter of the pMCB17apx vector was replaced with the amyB promoter as a EcoRI-KpnI fragment.

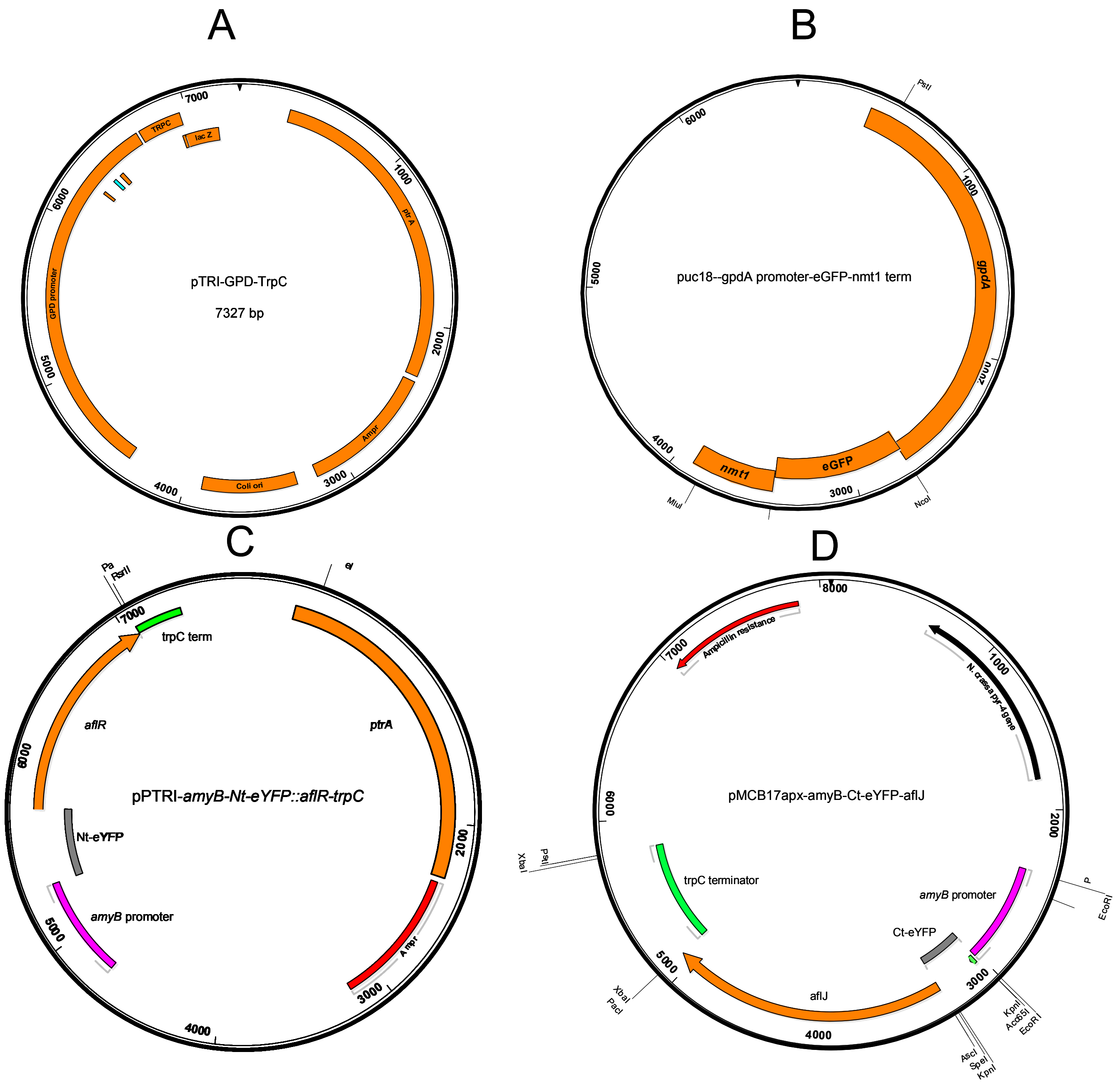

Figure S2. Thin layer chromatography results (various transformants TLC of extracts of mycelia from *A.parasiticus* SRRC2043Δ*aflJ-aflJ::GFP* transformants (accumulates OMST). (**A**) Δ*aflJ* produces no OMST whereas the gpd::aflJ::GFP complemented mutant produces OMST in quantities that approach those of the parental strain (RHN1 = AP2043 Δ*niaD*) in the Figure; (**B)** When aflJ is the promoter the yield of OMST is barely visible when grown in two media conducive for OMST expression. On Czapek's medium, *gpd-aflJ::GFP* does not complement the deletion as expected; (**C**) Δ*aflJ* complemented with wild-type *aflJ* and *aflJ* fused to different fluorescent tags.

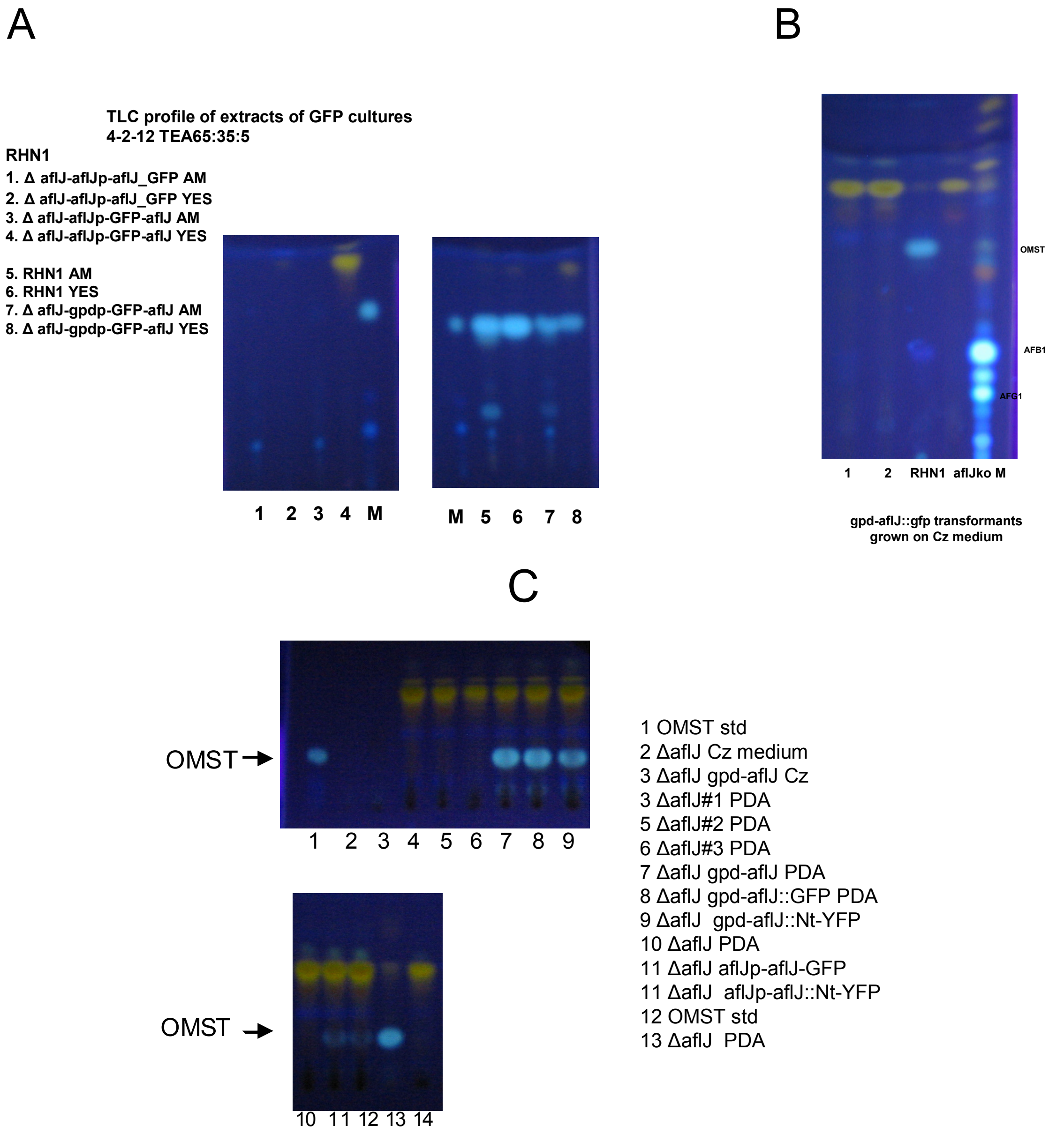

Figure S3. Sequence of *Dothistroma septosporum aflJ* (Provided by Dr Rosie Bradshaw).

DNA sequence

>aflJdothistroma-genomic(1-1482)

ATGTCGCGGATCTCTCGGCTGGGTGCATGCGCGGAGGAGCTTGCCATCGCTGCCACGACC
ATTGCGGCATTCTGCAAACACCATAGCAATTCTGGCCTACCTGGCGATAGCATACCGCCG
GACGCTCCCCAGAAGGTGCTGCAGGCCAAGCAATCCGTCATCACCAACTCACAGAAATTA
GAAGTGTTGCTGGCGGAACCGGCGGACTTTATACAACGTCTTGCGCGAGAGGTTCGCTCA
CAAACCCAATATGTCCATTTGCCGTCCATACTGATTGTGCTGTTCAGAACCAACTGCTGG
CTTGCTTGCAATGGCTCGGCGAGTTCCAAGTGCTTGCCTGCATTCCTATCGTGGATTCCG
TACACTACAGCGACGTGGCCGACCTTGCTTGCGTACCGGTCGATCAGCTGCGACGAATTG
CTCGCATGACAATCACGGCAGGCTTCCTCCAAGAGCCGAAGCCAGGGTATGTCGCTCACA
GCGGACTGTCGGCGCCGTTCGTAAAACAGCCCGTGCTGCTAGATGCAGCGATGTTCTTGT
CCGAGACCCTCGCGCCGTCTGCTCTTCACATGTCACTAGCGACGAAGCGCCATGGTCGAA
CTCACCAGACTGACCAATGCGCGTTCAATACCGCATTCAATACCAAGGCCAGTTTCGCGG
ATTCACTCGGACGAAGGCCTAGGCTGCAACGTCAATGGCCATCATTCTCAAGATACGCCA
TCGCCGATGACGAGGCTGGCGTCGAAGATGTTATGACTCGCTTGGACTGGCTTAGCCTAG
GCGAAGCTACAGTGGTCGATGTAGGTGCTGTGCCGAACAGTCATTAGGTCTGAGTACCCC
ATTGATCACGATCTACAGGTCTGTGCGAAGACAGCATCTCTCGCGACGGCACTGACGAGC
AAATATCCGTCGCTGCGGTTCGTCGTTCAAAGTGAAGAACAGTGCCAGAACCATACTTGG
TCGCGATCATTGTCAGCGACAAAGCTGCACAATGGTTTGTCGACACCTCCCGAATCCGAT
ACCGGACCCGCTGCACGGGCTGCCAAGGCAAGTGAACGTCTTGAGCTGCAACAACGAGCG
TTGGGCTCGCCGCAGAATGTCACCAATGCAGCCGTCTACATTCTGCGACTCGGTACAGCC
TCGCCTTTCACGTCCTGGCACAAGCTCAGAGCGCAGGCTACAGCAGAGCTCAGCGCTCAT
GCTGACATCTTGCGCAAAGAGCATGGATCAAGACTCATCCTGGTGACCCGCACTTTGCCG
AAGCCTGGCGAGGTAGAGACCACTGTCGAGGCCATGGCACGATTTCGAGACCTCACCCTG
ATGCAGCTGGCCAACGTAAGGGAACTGGAAACTTCCGAAGTCGTGGAATTATTGAACAGC
GTCCACATCGAGGGCGGATGCCTCGTGCTCACGAACGAGCTGAGAACCAGAAACAGCGGT
ATGATCGCATTCGAGGCGACTTATCAGCCGCAGCTCTTAGGA

Protein sequence

MSRISRLGACAEELAIAATTIAAFCKHHSNSGLPGDSIPPDAPQKVLQAKQSVITNSQKLEVLLAEPADF
IQRLARENQLLACLQWLGEFQVLACIPIVDSVHYSDVADLACVPVDQLRRIARMTITAGFLQEPKPGYVA
HSGLSAPFVKQPVLLDAAMFLSETLAPSALHMSLATKRHGRTHQTDQCAFNTAFNTKASFADSLGRRPRL
QRQWPSFSRYAIADDEAGVEDVMTRLDWLSLGEATVVDVCAKTASLATALTSKYPSLRFVVQSEEQCQNHTWS
RSLSATKLHNGLSTPPESDTGPAARAAKASERLELQQRALGSPQNVTNAAVYILRLGTASPFTSWHK
LRAQATAELSAHADILRKEHGSRLILVTRTLPKPGEVETTVEAMARFRDLTLMQLANVRELETSEVVELL
NSVHIEGGCLVLTNELRTRNSGMIAFEATYQPQLLG*

Figure S4. Sequences of *A. nidulans aflJ* homologs.

A. nidulans AN_10021

Genomic sequence

>ANID_10021

ATGTCTAGTCTATCCGACCTTGAAACCCACGCCAGTGAGCTCACAAGCGCTGTCAAGACG
ATCATCTCGCAATGCCCTCGCCAAAATGCCGCCTCTCGCAGCAGAACTCAACCCCTCATC
ACCTCTAGCGCTTCCAAGGAAGCGCATCGAGCCCAACAATCGATCTTATCAACCATTTCT
GGCCTCCAGAAGCTCCTCACCAGCCCAACCGACTTCCTCCACCACCTCGCCGTTCAGAAC
CAGCTGCTTGCCTGCCTACAATGGCTCGGAGAGTTCCAAGTCCTCGCTTGCATTCCCCTC
ACCGGCACCGTTCCCATAAAAGATGTCGCTGAGCTGGCCGGTGTCCCAGAGACTCATCTC
TCACGTATTATCCGGATGACAGCCACCGCTGGCTTCCTGGATGAGCCAGACCCCGGTCAA
GTCGCTCACAGCGCGCTCTCCGCTCCTTTCGTCACCAAACCGTCTTATCTTGACGCTGTG
ATGTTTTTGGCTGGCACCATTGCCCCTTCTGCTTTGCAGATGCCTACTGCAACGCAGCGA
TTTGGCGCGAGTTTGCGTCCGAACGAGACCGCGTACAACCTAGCTTTAAATAACCCAGCG
ACATTCGCCAGTACGTCTGAGCAACGGCCAAAGCTTCAACGCCAGTGGCCTGCTTTTCTT
CAGTATGGGACCAGTGATACCGACGATCGAGTGACGGATCTGTTGTCGAGGCTGGACCAT
TTTCGAAGAGGAAGTATATCTGTCGTTGAGGTACTTCATCCAATCCATCTTCATTTTGAG
ATCATCCATACGATTGTCTAACCAATTGCCCAAATTATAGGTCAGCGCCCGCTCCCTCGA
CCGCGCAACAACCCTTGCAAACCTCTACCCATCCATCAACATCACAGTCCAAATCGCATC
CCCAGCAGGCCCAACTGCCTGGTCACCAGCACACCCCAATCCCATCCGCCCCCCAACTCC
CGGCGGTAGCCACAAACACGACGACCTTCGCGCACTCACTGCAAGCACGGCCAGTACAAC
ACCGGCCTCTAGCCACAACCACACCCATACGCATACCACCAATAGCATACCCCAGGCCTC
CAACATAACGATCCAACACCGGCTTCCAACAGCACCGCAACCCATTACCTCAGCAAATCT
CTACATCCTACACCTCCCCTCTCCCTCACCAACAGTTCCTTTCGCCTCCCTTGCAACGCA
CATCCTCGCAGAACTCCGCTCACATCTCGACATCCTCCGCTCAAACCCATCGGCGACCCT
GATTCTCACCCCGCGGCCCTTGCCTGAACCCTCAGCTGTGCATAGCGAGGTCGAAGCAAG
CGCGCGACTGCGCGACTTGACGCTGATGCAGTTGGCAAATGAGCGTGAGATTGAGCTGGC
GGAGTGGATTAATCTGCTGAGCAATGTCAGTGATAGTATGGGCCGGTTGGTGGTGGTGAA
TAAGATTCAGTCCAGAGAAAGCACGGTAGTTTTGTTGGAGATTCGGTACCAGGCCTATAA
CAGGTGA

AN_10021 protein

MSSLSDLETHASELTSAVKTIISQCPRQNAASRSRTQPLITSSASKEAHRAQQSILSTIS
GLQKLLTSPTDFLHHLAVQNQLLACLQWLGEFQVLACIPLTGTVPIKDVAELAGVPETHL
SRIIRMTATAGFLDEPDPGQVAHSALSAPFVTKPSYLDAVMFLAGTIAPSALQMPTATQR
FGASLRPNETAYNLALNNPATFASTSEQRPKLQRQWPAFLQYGTSDTDDRVTDLLSRLDH
FRRGSISVVEVSARSLDRATTLANLYPSINITVQIASPAGPTAWSPAHPNPIRPPTPGGS
HKHDDLRALTASTASTTPASSHNHTHTHTTNSIPQASNITIQHRLPTAPQPITSANLYIL
HLPSPSPTVPFASLATHILAELRSHLDILRSNPSATLILTPRPLPEPSAVHSEVEASARL
RDLTLMQLANEREIELAEWINLLSNVSDSMGRLVVVNKIQSRESTVVLLEIRYQAYNR

Figure S5. CMEIAS: Definitions of measurement features.

(CMEIAS© Ver 1.28 [7] and http://cme.msu.edu/cmeias)

Major Axis Length: The maximum distance between points on the object's boundary.

Area: Area of the object, measured as the number of pixels (scaled to the user-defined unit for image calibration) in the polygonal approximation of the cell. This measurement of size tends to slightly overestimate the object's true area because the borders of the pixels may extend beyond the true perimeter of the cell.

Perimeter: Length of the outside contour of the object represented as a polygon in the digital image.

Roundness (also called "circularity" or "shape factor"): Computed as (4π Area/Perimeter^2). This shape feature measures the degree of object roundness. Values lie between 0 and 1. The greater the value, the rounder is the object.

Elongation: The ratio of the length of the major axis to the length of its minor axis. The result is a value ≥ 1. If the elongation is 1, the object is roughly circular or square. The ratio increases from 1 as the object becomes more elongated.

Compactness: Computed as:

$$\frac{\sqrt{4\text{Area}/\pi}}{\text{Major Axis Length}}$$

The feature measures the object's circularity, representing the ratio of the Feret diameter (defined below) to the object's major axis length, and ranges between 0 and 1. Objects with a compactness value of 1 are roughly circular.

Feret Diameter: Diameter of a circle having the same area as the object, computed as ($\sqrt{4 \mathit{Area}/\pi}$).

ABR: ratio of the object's area to the area of the smallest bounding box.

Figure S6. Additional eYFP fluorescence assays. Positive and negative controls for split eYFP studies. As a positive control a fusion protein was made with transformants expressing Nt-eYFP-veA + Ct-eYFP-laeA. In this control eYFP fluorescence was only detected in nuclei and not in organelles. The negative control used in the experiment was Nt-YFP-aflR + Ct-YFP-veA and in this case no fluorescence could be detected when grown on maltose-containing (inducing) medium.

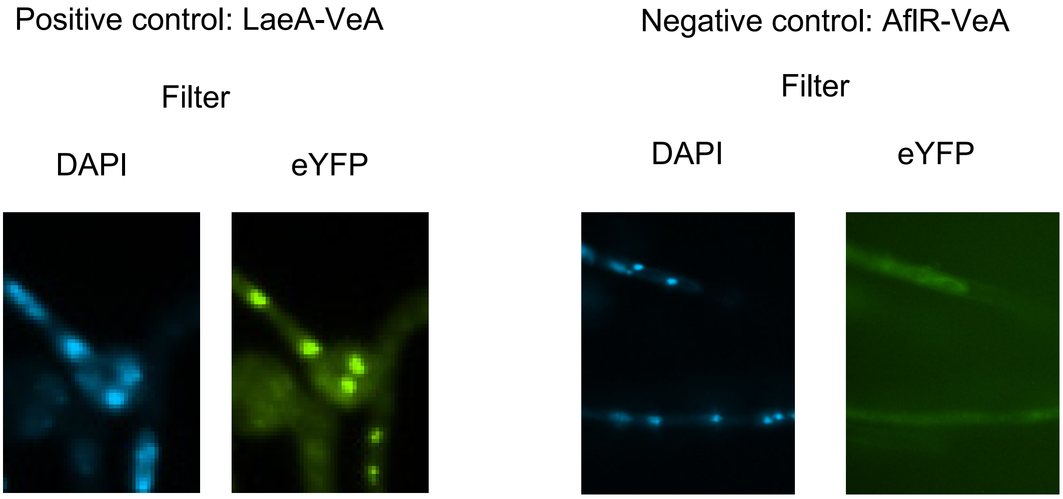

Figure S7. MDY staining of vacuoles in *A. parasiticus* Δ*aflJ* and wild-type. Cultures were grown on YES liquid medium for 68 h with shaking. Morphology was evaluated at indicated time points using bright field microscopy and fluorescent microscopy (Nikon). Mycelia were rinsed with sterile PBS, stained with MDY-64 (0.5 µL per slide). MDY-64 (Molecular Probes) is a green fluorescent dye that preferentially labels vacuolar membranes. Δ*aflJ* and wild-type both contained numerous medium size vacuoles (stained with MDY-64 and much smaller vesicles (not stained). In some cells vesicles that appeared to stain with MDY-64 had small vacuoles underlying the vesicles.

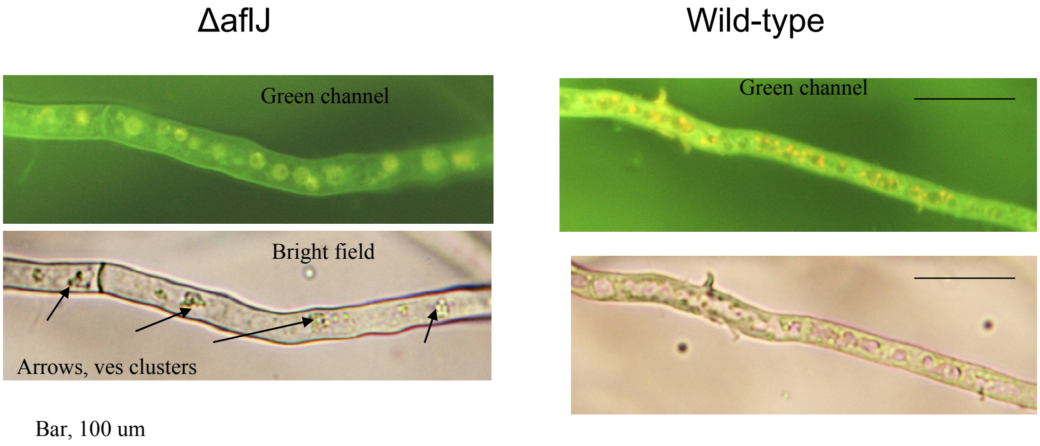

© 2012 by the authors; licensee MDPI, Basel, Switzerland. This article is an open access article distributed under the terms and conditions of the Creative Commons Attribution license (http://creativecommons.org/licenses/by/3.0/).
